# Fraturas extra-articulares da escápula

**DOI:** 10.1055/s-0046-1822982

**Published:** 2026-05-07

**Authors:** Vincenzo Giordano, Robinson Esteves Pires, Pedro José Labronici

**Affiliations:** 1 Serviço de Ortopedia e Traumatologia Prof. Nova Monteiro, Hospital Municipal Miguel Couto, Rio de Janeiro, RJ, Brasil; 2 Clínica São Vicente, Rede D'or São Luiz, Rio de Janeiro, RJ, Brasil; 3 Departamento do Aparelho Locomotor, Faculdade de Medicina, Universidade Federal de Minas Gerais (UFMG), Avenida Prof. Alfredo Balena, Belo Horizonte, MG, Brasil; 4 Hospital Felício Rocho, Belo Horizonte, MG, Brasil; 5 Departamento de Ortopedia e Traumatologia, Faculdade de Medicina, Universidade Federal Fluminense (UFF), Niterói, RJ, Brasil; 6 Serviço de Ortopedia e Traumatologia Prof. Dr. Donato D'Ângelo, Hospital Santa Teresa, Petrópolis, RJ, Brasil

**Keywords:** escápula, fixação de fratura, fraturas do ombro, fracture fixation, scapula, shoulder fractures

## Abstract

As fraturas extra-articulares da escápula incluem aquelas que acometem o processo coracoide, o acrômio, a espinha e o ângulo inferior da escápula. São lesões pouco frequentes, de modo que representam um desafio terapêutico. Historicamente, o tratamento não cirúrgico era considerado padrão para esses tipos de fraturas, embora os resultados fossem heterogêneos, frequentemente marcados por dor persistente, limitação da mobilidade e discinesia escapulotorácica. Atualmente, com a evolução dos métodos de imagem, das técnicas operatórias e da qualidade dos implantes, as indicações cirúrgicas têm se ampliado, mostrando bons resultados funcionais no médio e longo prazos. Neste artigo de atualização, os autores apresentam a literatura mais recente referente a cada um desses padrões de fratura da escápula e expõem as recomendações de tratamento.

## Abstract (en)

Extra-articular scapular fractures include those involving the coracoid process, the acromion, the scapular spine, and the inferior angle of the scapula. These injuries are uncommon, so they represent a therapeutic challenge. Historically, nonoperative treatment was the standard for these fracture types, although the outcomes were heterogeneous and often characterized by persistent pain, limited mobility, and scapulothoracic dyskinesis. Advances in imaging, surgical techniques, and implant quality have expanded surgical indications, demonstrating good medium- and long-term functional outcomes. In the current update article, the authors present the most recent literature on each of these scapular-fracture patterns and outline the current treatment recommendations.

## Introdução

As fraturas da escápula são raras, e representam menos de 1% de todas as fraturas e aproximadamente 5% das fraturas da cintura escapular. 1 2 Com relação a essa baixa incidência, as fraturas extra-articulares do processo coracoide, do acrômio, do corpo e da espinha da escápula representam a maioria dos casos, geralmente com desfechos funcionais favoráveis em cerca de 80% dos pacientes tratados de forma não cirúrgica. 2 3 Esse resultado é atribuído à grande amplitude de movimento do ombro, que permite a manutenção das atividades de vida diária mesmo diante de algum grau de deformidade pós-traumática. 3 Entretanto, queixas como dor persistente e limitação da mobilidade do ombro são observadas em alguns pacientes, e impactam de maneira significativa a função do membro superior acometido. Nesse contexto, torna-se necessária a definição de parâmetros mais específicos que permitam identificar de forma adequada quais fraturas extra-articulares da escápula se beneficiaram do tratamento cirúrgico.

Atualmente, sobretudo em razão dos avanços nos exames de imagem, que têm facilitado a caracterização morfológica e aumentado a acurácia na avaliação dos desvios, o tratamento cirúrgico vem sendo mais frequentemente indicado em determinados padrões de fratura do processo coracoide, acrômio, espinha e ângulo inferior da escápula. 2 4 Essas indicações são consideradas, em especial, para prevenir disfunção do complexo suspensório superior do ombro (CSSO), invasão do espaço subacromial, comprometimento do manguito rotador e/ou restrições da mobilidade escapulotorácica. 2 4 5 6 7

Neste artigo, revisamos as indicações cirúrgicas e apresentamos as principais abordagens e opções atuais de fixação para fraturas do processo coracoide, do acrômio, da espinha e do ângulo inferior da escápula.

## Fraturas do Acrômio

Fraturas do acrômio são raras, e representam cerca de 8% de todas as fraturas da escápula. 2 8 O mecanismo clássico de lesão é um trauma direto sobre a superfície lateral do ombro, que frequentemente ocorre em associação com outras lesões da cintura escapular e/ou do hemitórax ipsilateral. Mais raramente, as fraturas do acrômio podem ocorrer após acromioplastia, sendo mais observadas após cirurgia artroscópica do que o procedimento aberto, ou como uma complicação pós-operatória de artroplastia total reversa do ombro (ATRO), em decorrência da alterações biomecânicas e do comprimento do braço. 2 8 9 O diagnóstico por vezes é difícil, e exige alto grau de suspeição por parte do cirurgião sempre que o paciente queixar-se de dor intensa no ombro após um trauma direto na face lateral do ombro. O estudo radiográfico deve incluir a incidência axilar, considerada a mais sensível para a detecção da fratura, e a tomografia computadorizada (TC), incluindo a reconstrução tridimensional (3D TC). Em alguns casos, como os das fraturas por insuficiência após ATRO, uma imagem de ressonância magnética pode ser necessária para visualizar melhor a fratura. É importante ter em mente que o *os acromiale* , presente em cerca de 3% da população, pode confundir-se com uma linha de fratura. 8

O tratamento das fraturas do acrômio permanece controverso na literatura atual, não havendo indicações cirúrgicas absolutas. A anatomia singular, complexa e delgada dessa estrutura, associada às suas múltiplas inserções ligamentares e musculares, torna o manejo particularmente desafiador. 2 A maioria dos autores recomenda redução anatômica e fixação interna para fraturas com ≥ 1 cm de desvio radiográfico, fratura ipsilateral da escápula com indicação cirúrgica, ou em casos de lesão múltipla do CSSO, com o objetivo de prevenir a pseudartrose dolorosa e proteger o manguito rotador de impacto subacromial. 2 7

A adoção de um sistema de classificação auxilia na tomada de decisão terapêutica. Entre as classificações, a de Kuhn et al. 10 é tida como a mais útil, pois considera tanto o desvio do fragmento quanto a redução do espaço subacromial.

Segundo esse sistema, o tratamento não cirúrgico está indicado para a maioria das fraturas estáveis e não desviadas (tipo I de Kuhn) e para fraturas desviadas sem redução do espaço subacromial (tipo II de Kuhn) em pacientes de baixa demanda funcional. Esses casos exigem acompanhamentos clínico e radiográfico rigorosos, devido ao risco de desvio tardio e progressivo, especialmente nos tipos IB e II. 10 O tratamento recomendado consiste no uso de tipoia simples por 6 a 8 semanas, iniciando a mobilização passiva assistida a partir da terceira semana, e a ativa assistida, após a sexta semana. 11

Embora em teoria o uso de uma tipoia de abdução possa reduzir o braço de alavanca do músculo deltoide, e assim diminuir o risco de desvio secundário da fratura, não há evidências clínicas que sustentem sua superioridade em relação à tipoia simples. 2 8 12 A remoção da tipoia deve ser feita após existirem sinais radiográficos ou tomográficos de consolidação da fratura. 11 Nos demais tipos de fratura, incluindo o tipo IB em pacientes de alta demanda, o tratamento cirúrgico está indicado. 10 11 A abordagem cirúrgica deve ser feita seguindo as linhas de força de Langer, preferencialmente logo acima da espinha da escápula, como uma incisão horizontal posterolateral. 13 Se houver fratura associada da fossa ou do colo da glenoide ou do corpo da escápula, a abordagem de Brodsky com extensão proximal em curva é uma alternativa útil. 2 Quando há a necessidade de uma exposição mais ampla, com extensão medial, deve-se identificar e proteger o nervo supraescapular.

**Fig. 1:**
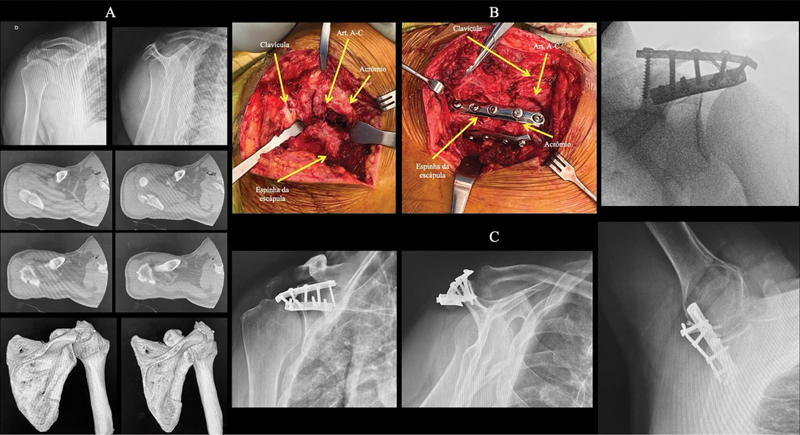
Fratura do acrômio direito do tipo III de Kuhn et al. 10 ( **A** ) Radiografias e cortes tomográficos que evidenciam a fratura e a invasão do espaço subacromial; ( **B** ) Imagens intraoperatórias da fratura e da fixação com dupla placa; ( **C** ) Radiografias pós-operatórias com 1 ano, que mostram fratura consolidada em boa posição e função normal do ombro.

Embora várias opções de fixação tenham sido descritas para o tratamento das fraturas do acrômio, a preferência recai sobre o uso de dupla placa ortogonal, com implantes bloqueados e de baixo perfil 11 ( Fig. 1 ). Diversos autores 2 7 8 13 14 15 têm demonstrado bons resultados com esse método, e destacam a baixa taxa de complicações e de necessidade de retirada dos implantes. Nas fraturas muito distais, nas quais o uso de placas não é viável, a técnica de banda de tensão deve ser considerada, pois permite adequado controle rotacional do fragmento. 7 8 13 Nesse padrão de fratura, o uso isolado de parafusos deve ser evitado, uma vez que não neutraliza as forças deformantes exercidas pelo músculo deltoide e pelo próprio peso do membro superior. 11 Antes do fechamento da ferida cirúrgica, é fundamental realizar controle radiográfico, incluindo a incidência anteroposterior com 30° de inclinação caudal (incidência de Rockwood), a fim de garantir que não haja penetração do espaço subacromial pelos parafusos da osteossíntese. 16

No pós-operatório, iniciam-se exercícios passivos e ativos assistidos conforme a tolerância, mantendo abdução e flexão limitadas a 50°. Utiliza-se tipoia simples por 1 a 2 semanas. A mobilidade é progressivamente ampliada nas primeiras 6 semanas, com controles radiográficos na primeira, terceira e sexta semanas. A partir da sexta semana, iniciam-se exercícios isométricos com elásticos de baixa resistência. 11

## Fraturas do Coracoide

As fraturas do coracoide representam aproximadamente 3 a 13% de todas as fraturas da escápula. 2 7 11 17 18 São em geral causadas por traumas de alta energia, e estão frequentemente associadas a lesões complexas em outras partes da própria escápula e em estruturas adjacentes, em especial a articulação acromioclavicular, a região lateral da clavícula e a proximal do úmero. 19

No entanto, menos frequentemente, e muitas vezes não diagnosticadas no primeiro atendimento, as fraturas do processo coracoide podem ocorrer por trauma indireto, especialmente após luxação anterior do ombro. 19 Fraturas por estresse representam causa ainda mais rara, embora tenham sido descritas em modalidades esportivas como tiro, críquete e golfe. 19

A visualização radiográfica do processo coracoide é dificultada por sua orientação tridimensional complexa e variável, de modo que as incidências tradicionais da série trauma do ombro podem não permitir diagnóstico preciso. 18 19 20 Por essa razão, além da série trauma, recomenda-se a obtenção de radiografias ortogonais específicas para o pilar coracoide, como a incidência descrita por Bhatia, que aprimoram a identificação de fraturas dos pilares superior e inferior, bem como da região juncional do processo coracoide. 11 20 Para essas incidências o paciente é posicionado em pé ou em decúbito dorsal, com o cassete apoiado sobre o aspecto posterior do ombro afetado.

A visão do pilar coracoide inferior (PCI) é obtida com inclinação cefálica de 30° a 40° do feixe radiográfico, alinhando-o perpendicularmente ao plano sagital do processo coracoide. Adicionalmente, aplica-se uma angulação no plano axial de lateral para medial (20°–30°), o que centraliza o feixe sobre a ponta do coracoide.

A visão do pilar coracoide superior (PCS) é obtida inclinando-se o ombro afetado 30° posteriormente, posicionando a escápula paralelamente ao cassete, posição semelhante à incidência de Grashey. O feixe é angulado de 30° a 40° cefalicamente, e de 20° a 30° de medial para lateral no plano axial, centralizado sobre a ponta do coracoide. 11 20 Além das radiografias, a avaliação deve obrigatoriamente ser complementada por TC, incluindo reconstruções 3D e subtração óssea das estruturas adjacentes. 17

Na tomada de decisão terapêutica, recomenda-se a utilização dos sistemas de classificação de Bartoníček et al., 17 que considera a posição anatômica da fratura, e de Ogawa et al., 19 21 que se baseia na relação entre o traço de fratura e o ligamento coracoclavicular. 11

Nas fraturas isoladas e não desviadas, ou minimamente desviadas, do processo coracoide, o tratamento não cirúrgico pode ser indicado com bons resultados, até mesmo quando a conformação do arco coracoacromial apresenta discreta alteração. 21 Em uma revisão sistemática de 97 estudos, 19 que incluiu 197 pacientes, 71% das fraturas isoladas classificadas como de tipo I por Ogawa foram manejadas de forma não cirúrgica, com desfechos favoráveis até mesmo nos casos de não consolidação.

Nesses casos, recomendamos o uso de tipoia simples por 6 a 8 semanas, iniciando mobilização passiva assistida a partir da terceira semana e mobilização ativa assistida após a sexta semana. 11 Deve-se evitar flexão ativa do ombro e do cotovelo ipsilaterais no período inicial, pois a tração exercida pela cabeça curta do bíceps pode desviar o fragmento. A retirada da tipoia deve ocorrer somente após confirmação radiográfica ou tomográfica de consolidação.

As indicações para tratamento cirúrgico incluem fraturas localizadas proximalmente ao ligamento coracoclavicular, desvio ≥ 1 cm na avaliação por imagem ou presença de lesão múltipla do CSSO. 2 7 17 21 O objetivo do procedimento é preservar a configuração do arco coracoacromial e manter a continuidade entre clavícula e escápula.

A maioria das fraturas do coracoide pode ser abordada por uma incisão vertical direta, seguindo as linhas de Langer, iniciada no aspecto superior da clavícula, imediatamente acima do intervalo coracoclavicular. 22 Nos casos associados a fraturas da glenoide anterior ou superior, a abordagem deltopeitoral de Henry é preferida. 2 7 De forma menos comum, algumas fraturas do tipo I de Ogawa não desviadas podem ser fixadas por via percutânea, com ou sem assistência artroscópica. 2

Durante a redução da fratura, o membro superior operado deve ser colocado em rotação interna e adução para proteger o plexo braquial. 2 Suturas aplicadas ao tendão conjunto e fios rosqueados posicionados ortogonalmente ao fragmento auxiliam na mobilização deste, o que facilita a redução. 2 7 11 Após a redução, utiliza-se um fio Kirschner para fixação temporária. 11

**Fig. 2:**
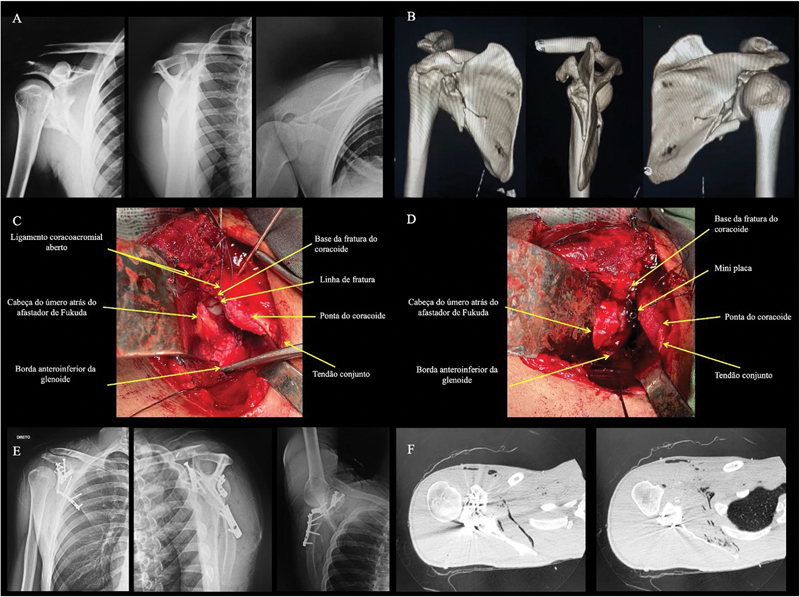
( **A** , **B** ) Radiografias da série trauma e tomografia computadorizada tridimensional (TC 3D) do ombro direito, que mostra fratura complexa da escápula, com envolvimento do processo coracoide. A fratura do coracoide foi classificada como do tipo III (base) de Bartoníček et al., 17 e do tipo II (distal à inserção do ligamento coracoclavicular) de Ogawa et al.; 19 21 ( **C** , **D** ) Imagens intraoperatórias que evidenciam a linha de fratura após a abertura do ligamento coracromial e após sua fixação com uma placa de minifragmento de 2,0 mm e um parafuso de tração de 3,5 mm. O ligamento coracoacromial foi reparado usando-se parafuso âncora depois que a fratura do coracoide foi fixada; ( **E** , **F** ) Radiografias da série trauma e cortes axiais de TC do ombro direito realizados após a cirurgia, que mostram a fixação de todos os traços de fratura da escápula e a qualidade de redução do coracoide e da glenoide.

A fixação definitiva é geralmente realizada com um parafuso cortical de 3,5 mm ou, menos frequentemente, com um parafuso canulado parcialmente rosqueado de mesmo calibre. 2 11 A correta orientação do parafuso é essencial para garantir estabilidade e evitar falhas. Como a ponta do coracoide é fina e curva, em formato de gancho, a fixação iniciada por esse ponto é tecnicamente difícil; por isso, o posicionamento ideal é no corpo do coracoide, em direção à sua base e ao colo da escápula, trajeto conhecido como *túnel coracoide* . 2 11 17 23 ( Fig. 2 ).

No pós-operatório, são iniciados exercícios passivos e ativos assistidos, limitando a abdução e a flexão maior que 50° e evitando flexão contra a resistência do cotovelo. Utiliza-se tipoia simples por 1 a 2 semanas para conforto. A amplitude de movimento é aumentada gradualmente nas primeiras 6 semanas, com acompanhamentos clínico e radiográfico na primeira, terceira e sexta semanas. Após a sexta semana, iniciam-se exercícios isométricos com elásticos leves. 11

## Fraturas da Espinha da Escápula

As fraturas da espinha da escápula são relativamente raras, e correspondem a menos de 11% de todas as fraturas da escápula. Embora o mecanismo de lesão seja tipicamente associado a traumas de alta energia, fraturas da espinha têm sido observadas com maior frequência como complicação da ATRO. 5 24 25 26 A incidência global de fraturas do acrômio e da espinha associadas à ATRO varia entre 0,8% e 10,2%. 5 24 25 26 Neste artigo, fraturas secundárias à ATRO não serão abordadas por apresentarem etiologia e conduta distintas das traumáticas. 25

Nos traumatismos de alta energia, as fraturas da espinha raramente ocorrem de forma isolada, estando geralmente associadas a fraturas do corpo ou da glenoide. 11 23 Nesses casos, um elevado índice de suspeição é fundamental, devendo ser valorizados, além da dor local e à mobilização, sinais de disfunção do membro superior ipsilateral. 23

**Fig. 3:**
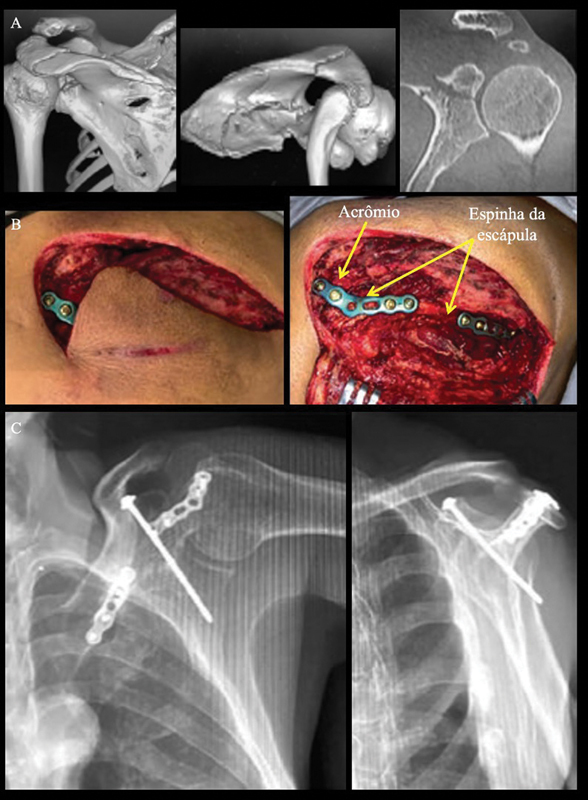
( **A** ) Tomografias computadorizadas 3D e bidemensional (2D) coronal do ombro esquerdo, que mostram fratura segmentar da espinha da escápula e da base do processo coracoide com extensão à fossa glenoide. Observe o formato em S horizontal da espinha; ( **B** ) Imagens intraoperatórias que evidenciam a fratura segmentar da espinha da escápula e a fixação com placas bloqueadas dorsais de 2,4 mm; ( **C** ) Radiografias em cisões anteroposterior (AP) e de perfil da escápula esquerda realizadas após a cirurgia, que mostram a fixação das fraturas da espinha da escápula e do processo coracoide com um parafuso de tração de 2,5 mm. Note a qualidade de redução do coracoide e da fossa glenoide.

O tratamento é predominantemente cirúrgico, e a posição do paciente e a via de acesso dependem das fraturas associadas da escápula. 11 23 O tratamento costuma ser cirúrgico, e a escolha do posicionamento do paciente e da via de acesso depende das fraturas associadas da própria escápula. 11 23 O paciente pode ser colocado em decúbito lateral, contralateral ou em decúbito ventral, utilizando-se miniabordagens direcionadas a cada tipo de fratura. A fixação da espinha é preferencialmente realizada com implantes bloqueados de menor diâmetro (2,0–2,7mm), podendo-se empregar placas de pequenos fragmentos (3,5 mm) quando a espessura óssea for suficiente 27 28 ( Fig. 3 ).

No pós-operatório, utiliza-se tipoia simples por 6 semanas. Iniciam-se exercícios passivos e ativos assistidos conforme tolerados, limitando abdução e flexão a 50°. Os exercícios de amplitude são progressivamente aumentados ao longo das 6 primeiras semanas. Os seguimentos clínico e radiográfico são realizados na primeira, terceira e sexta semanas. A partir da sexta semana, iniciam-se exercícios isométricos com elásticos de baixa resistência. 11 23

## Fraturas do Ângulo Inferior da Escápula

A fratura isolada do ângulo inferior da escápula é rara e geralmente ocorre em traumas de menor energia, por avulsão dos músculos periescapulares, como o serrátil anterior, redondo maior, romboide maior e grande dorsal. 29 30 Em pacientes mais idosos, nos quais a fratura ocorre devido à osteoporose acentuada, é necessário alto grau de suspeição diagnóstica. Nesses casos, radiografias do tórax e da série trauma do ombro frequentemente não evidenciam a fratura, sobretudo quando não há desvio inicial evidente. Assim, a TC é fundamental em pacientes com dor intensa localizada, equimose tardia ou disfunção persistente por tempo superior ao esperado para uma lesão inicialmente presumida como muscular. 30 31

Na maior parte dos casos, entretanto, a fratura do ângulo inferior está presente no contexto de uma fratura multifragmentar do corpo da escápula decorrente de trauma de alta energia. 4 30 Lesões associadas no hemitórax ipsilateral e na cintura escapular são comuns, incluindo paralisia do nervo torácico longo, que pode evoluir com escápula alada independentemente do tratamento instituído. 4 31 32 Em uma revisão sistemática de 17 artigos, Mousafeiris et al. 32 observaram desvio anterior em 64% dos casos, e escápula alada na mesma proporção.

Nas fraturas isoladas do ângulo inferior sem desvio ou com desvio mínimo, e sem angulação anterior, o tratamento é preferencialmente não cirúrgico, com analgesia e uso de tipoia simples por 6 a 8 semanas, mas realizando exercícios pendulares sem elevação do membro. 4 Avaliações clínica e radiográfica devem ser realizadas a cada 2 semanas para monitorar possível desvio secundário. A partir da sexta semana, inicia-se a liberação gradual para aumento da amplitude de movimento do ombro. 4 32

**Fig. 4:**
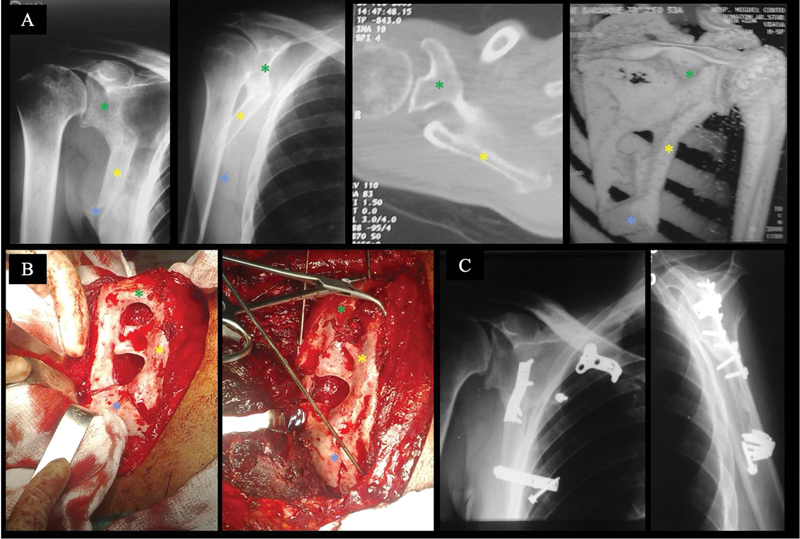
( **A** ) Radiografias em visões AP e de perfil de escápula, TC 2D axial e TC 3D do ombro direito, que mostram fratura complexa viciosamente consolidada do corpo da escápula. Observe o grau de deformidade e encurtamento. Clinicamente, o paciente apresentava discinesia escapulotorácica; ( **B** ) Imagens intraoperatórias que evidenciam a deformidade do corpo da escápula e os defeitos de consolidação. A fratura foi abordada pela via clássica de Judet, sendo realizadas múltiplas osteotomias e derrotação da borda lateral e do ângulo inferior; ( **C** ) Radiografias em AP e de perfil de escápula do ombro esquerdo realizadas após 10 anos da cirurgia. Observe a correção total do eixo do corpo. A fixação foi feita com placas de pequenos fragmentos. Nas imagens, os três fragmentos principais são destacados com asterisco verde (*) – fragmento superior, com o colo e a fossa da glenoide, processo coracoide, espinha da escápula e acrômio, fragmento lateral (asterisco amarelo), com a borda lateral da escápula, e ângulo inferior da escápula (asterisco azul).

Nas fraturas isoladas do ângulo inferior com desvio ou inclinação da borda medial, bem como nas fraturas do ângulo inferior associadas a outros traços no mesmo osso, está indicado o tratamento cirúrgico. 23 A via de acesso deve seguir as linhas de força de Langer, preferencialmente em orientação horizontal ou curvilínea no nível do ângulo inferior. 23 Se houver fratura associada do corpo da escápula, a escolha da abordagem, ou combinações de abordagens, deve ser definida conforme os traços a serem fixados 30 ( Fig. 4 ).

No pós-operatório inicial, utiliza-se tipoia simples por 7 a 10 dias, até a retirada dos pontos e controle adequado da dor, quando se iniciam exercícios passivos e ativos do membro superior ipsilateral. 4 23 31 32 33 Nos casos em que o tratamento não cirúrgico falha ou o diagnóstico inicial não é realizado, a consolidação viciosa com inclinação da escápula pode resultar em discinesia escapulotorácica. 34 35

## Considerações Finais

Devido à sua baixa frequência, as fraturas do processo coracoide, do acrômio, da espinha e do ângulo inferior da escápula continuam a representar um desafio para os cirurgiões que tratam lesões escapulotorácicas. Com os avanços na qualidade dos exames de imagem e na disponibilidade de implantes, algumas fraturas desviadas, anteriormente manejadas de forma não cirúrgica, passaram a ser tratadas cirurgicamente, com bons resultados relatados na literatura.

Essas indicações cirúrgicas, contudo, baseiam-se predominantemente em revisões sistemáticas com baixo nível de evidência, estudos retrospectivos de séries de casos e relatos isolados. Assim, as informações provenientes desses trabalhos devem ser aplicadas com cautela ao se estabelecer recomendações individualizadas, considerando sempre o perfil do paciente, sua demanda funcional, os recursos disponíveis na instituição e a experiência do cirurgião na abordagem dessas fraturas complexas.
